# Supplementary figures and images for: The hypoxia-associated genes in immune infiltration and treatment options of lung adenocarcinoma
Source: PeerJ. 2023 Aug 7;11:e15621. doi: 10.7717/peerj.15621 (PMC10414028; doi:10.7717/peerj.15621)

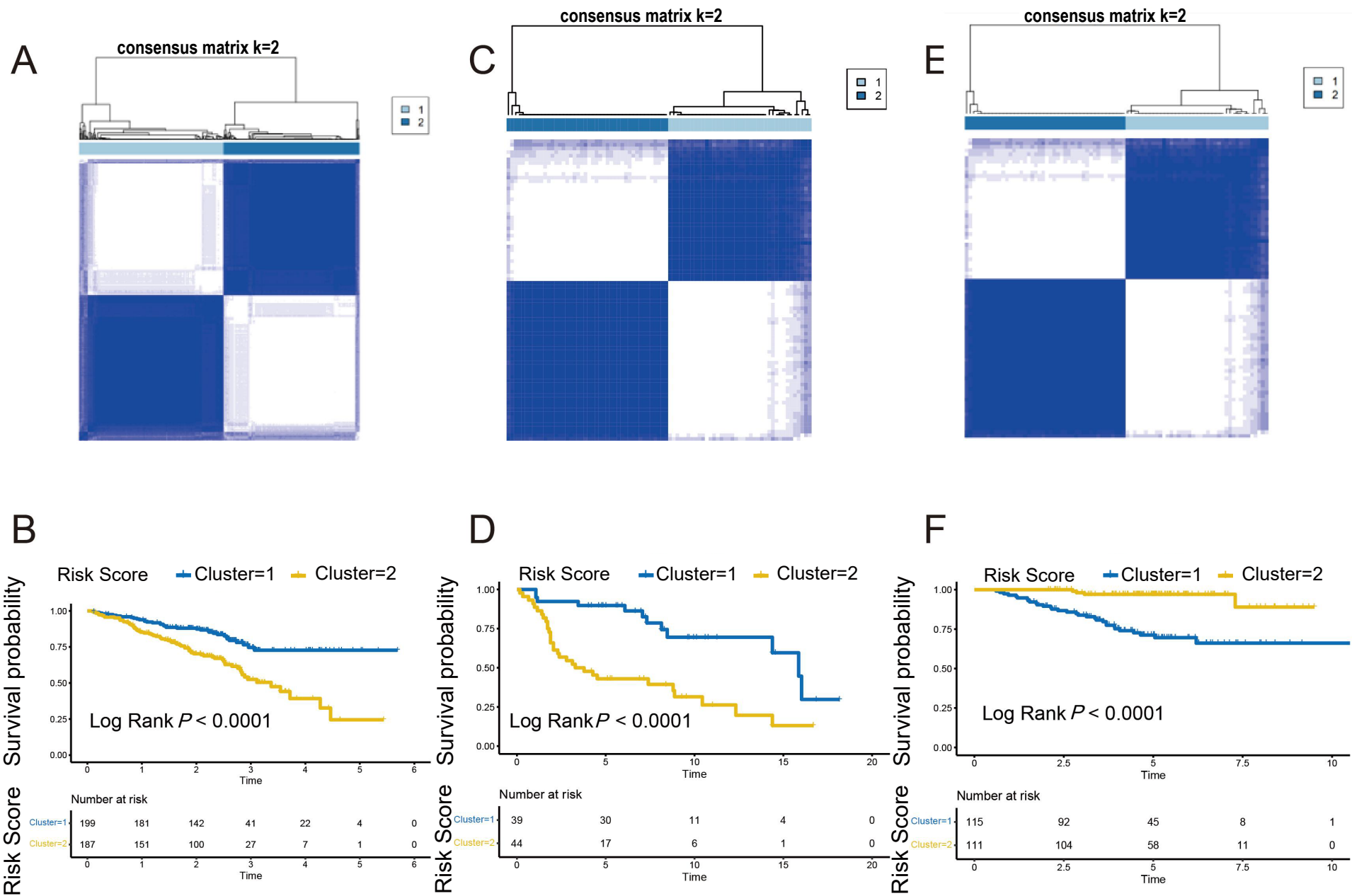

Supplement: Figure S1 [file peerj-11-15621-s001.pdf]

A

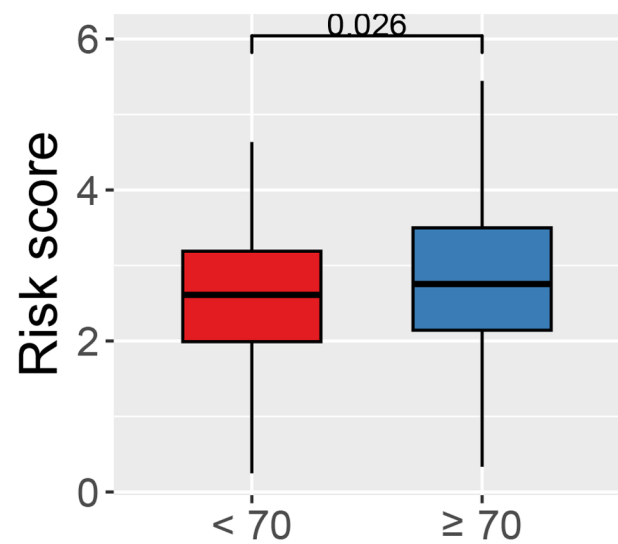

B

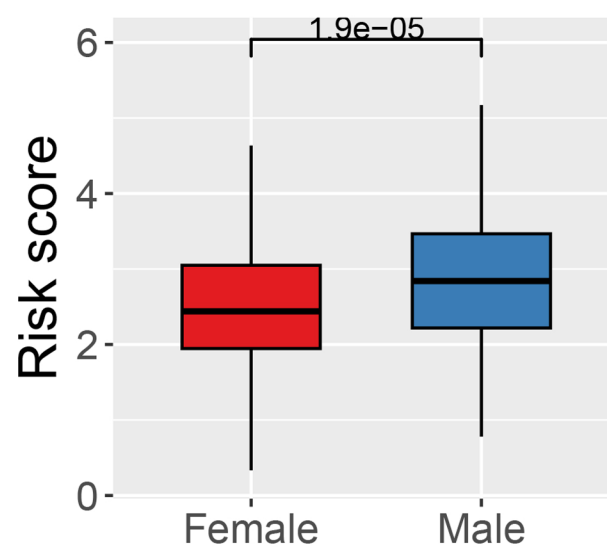

C

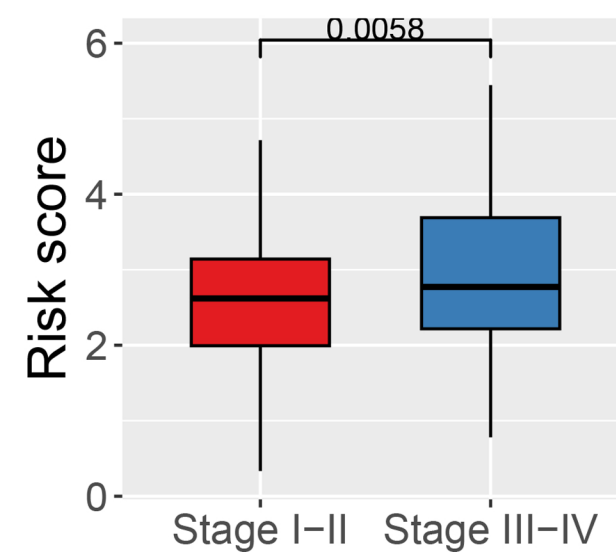

D

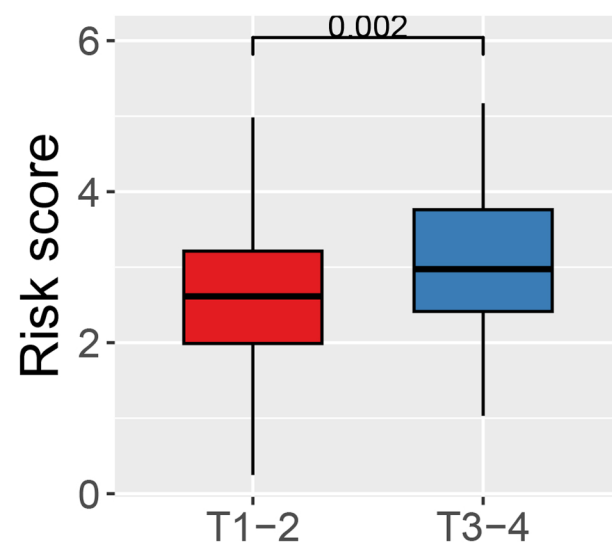

E

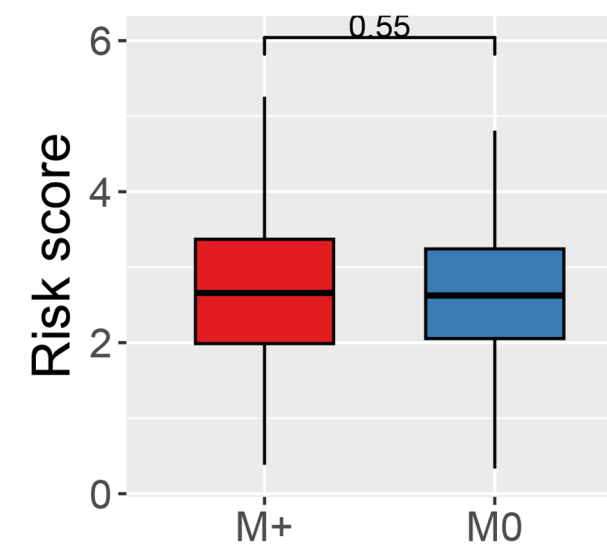

F

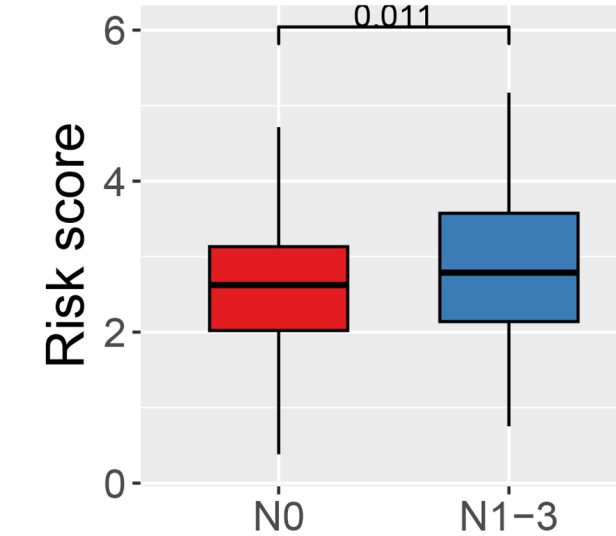

Supplement: Figure S2 — * p-value < 0.05, ** p-value < 0.01, *** p-value < 0.001. [file peerj-11-15621-s002.pdf]

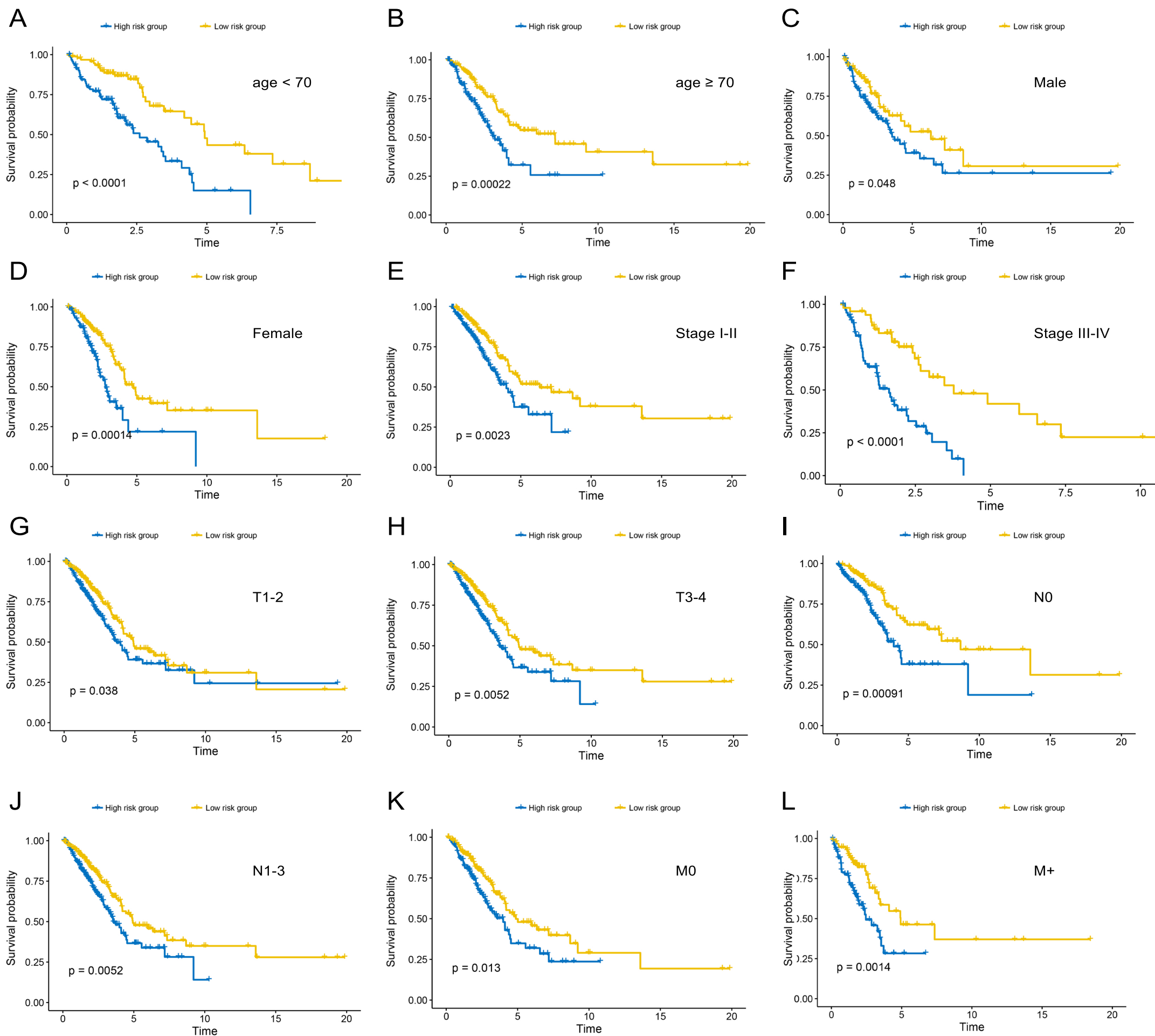

Supplement: Figure S3 — Kaplan-Meier curves for OS prediction in LUAD subtypes of (A) Age <70 years, (B) Age ≥70 years, (C) Male, (D) Female, (E) Pathological stage I-II, (F) Pathological stage III-IV, (G) Pathological T1-2, (H) Pathological T3-4, (I) Pathological N0, (J) Pathological N1-3, (K) Pathological M0, (L) Pathological M+. [file peerj-11-15621-s003.pdf]

A

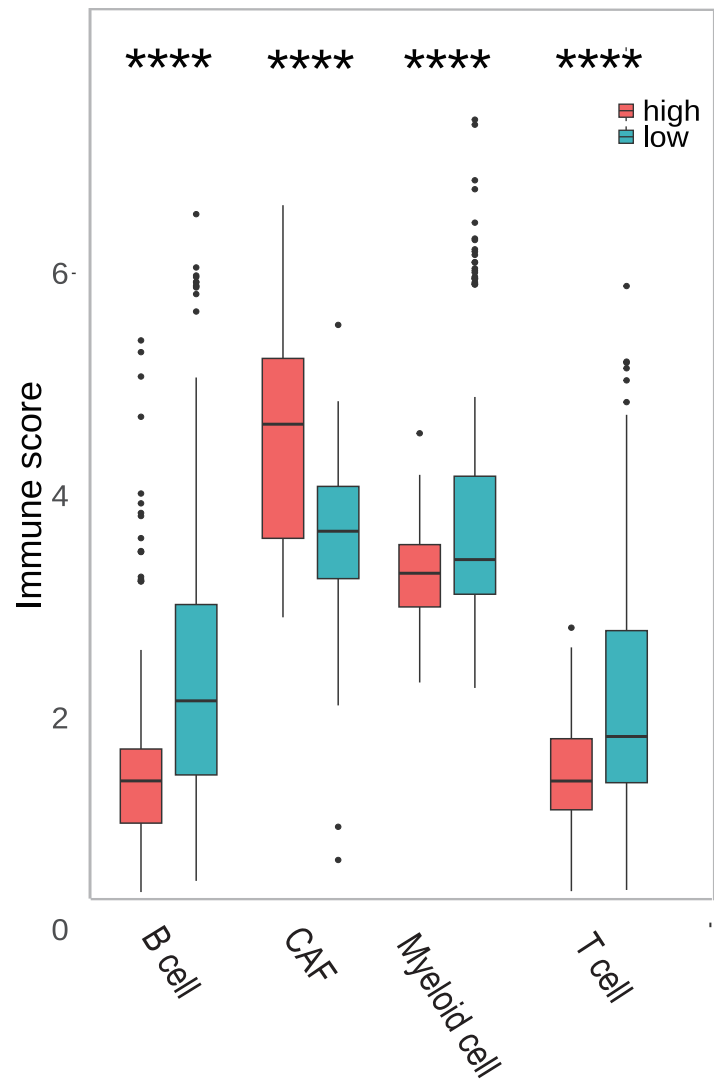

B

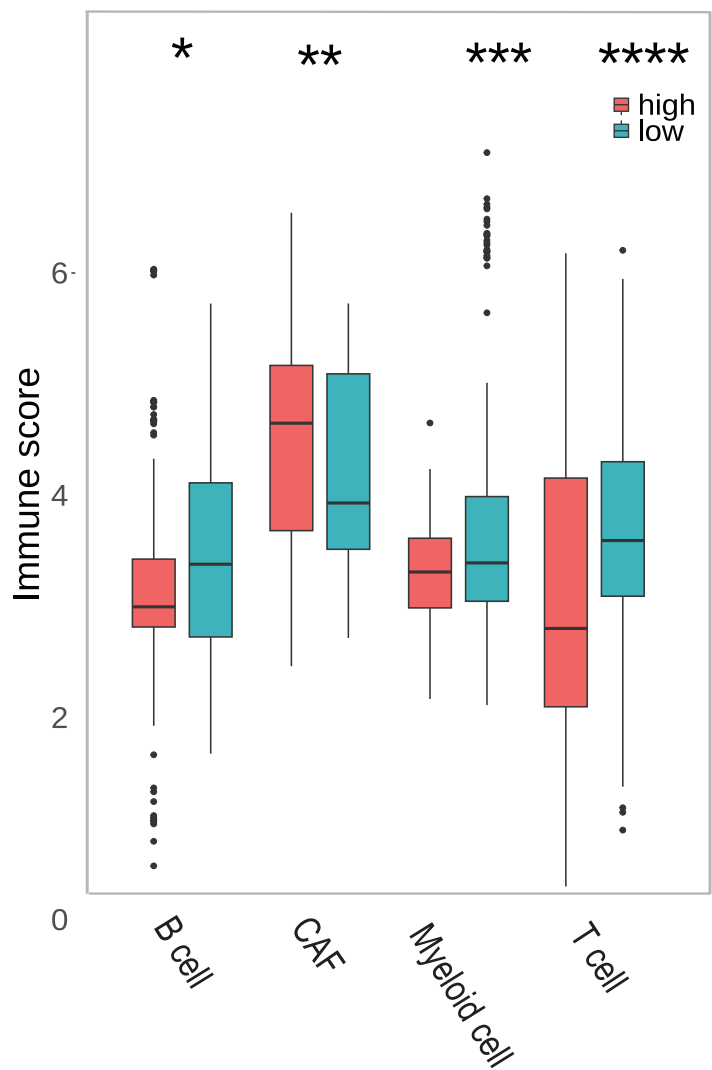

C

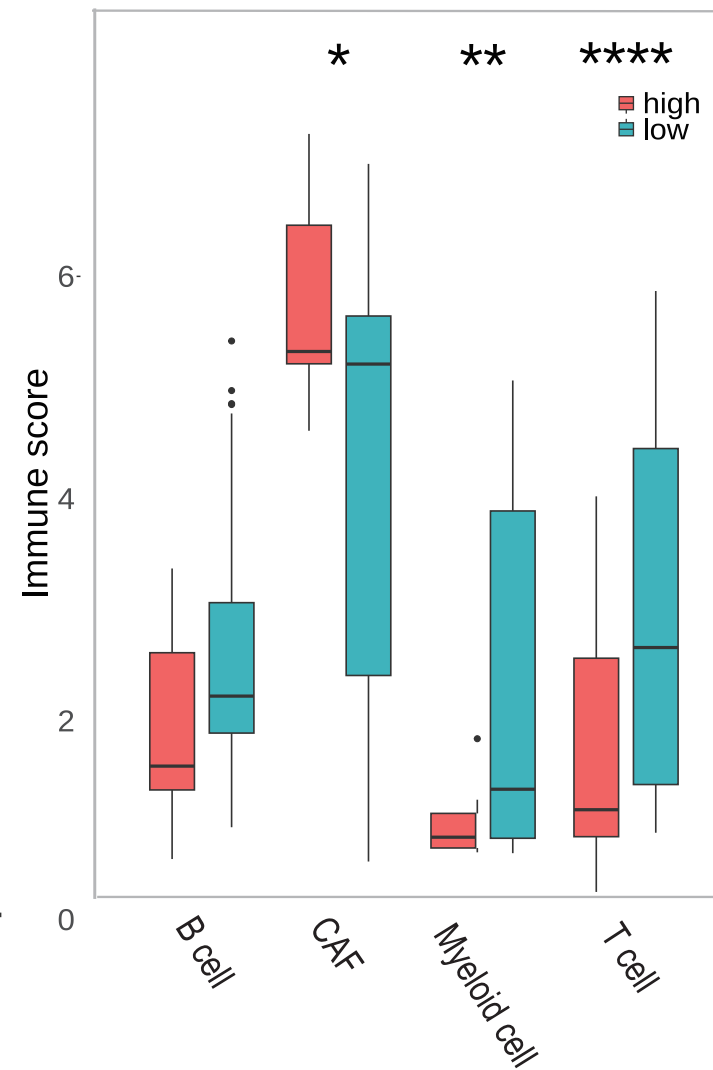

Tumor-infiltrating immune cells

Supplement: Figure S4 — GSE72094 (A), GSE31210 (B) and GSE30219 (C) cohorts. [file peerj-11-15621-s004.pdf]

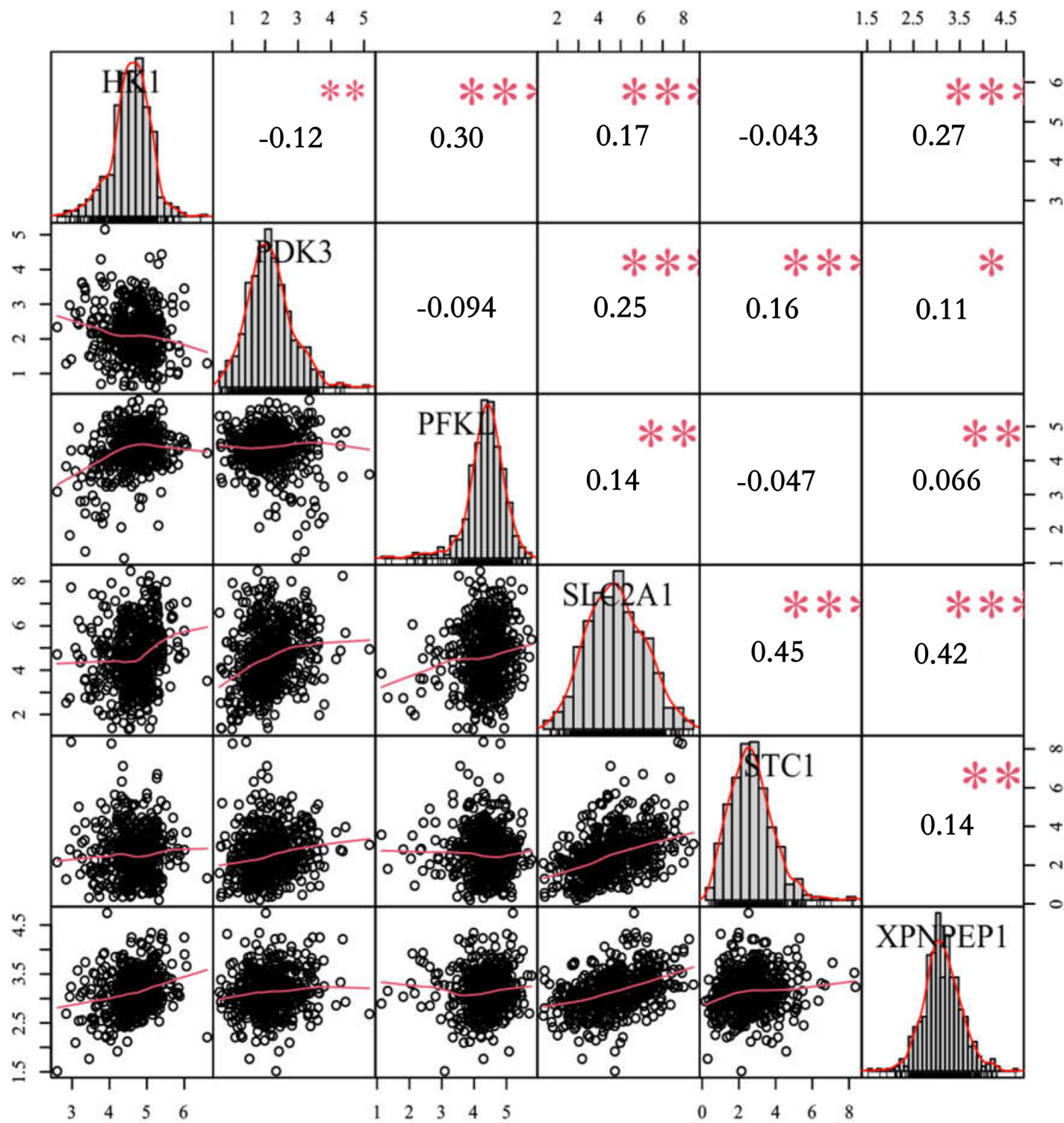

Supplement: Figure S5 [file peerj-11-15621-s005.pdf]

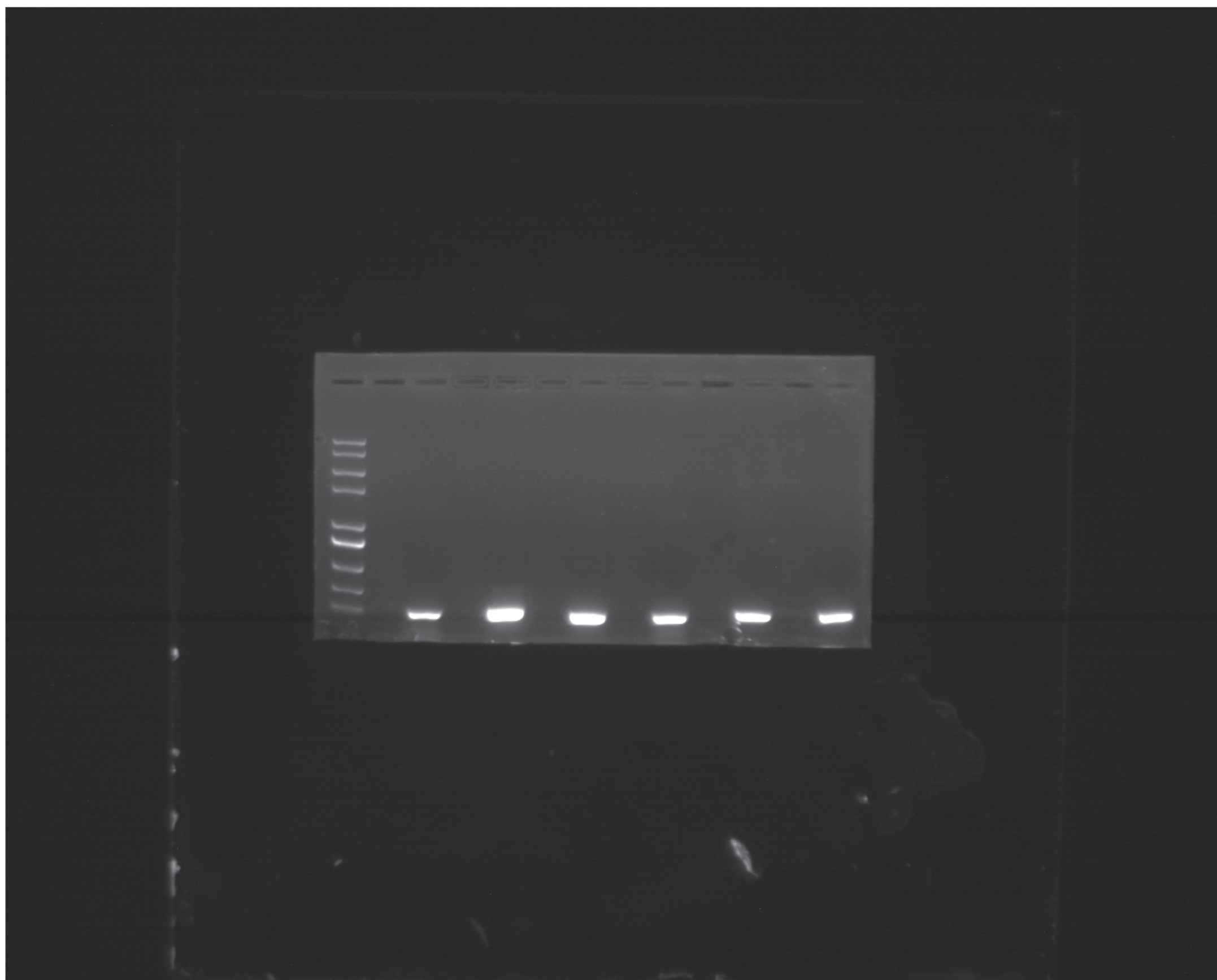

Supplement: Supplemental Information 8 [file peerj-11-15621-s008.pdf]
