# Supplementary material for: The hypoxia-associated genes in immune infiltration and treatment options of lung adenocarcinoma
Source: PeerJ. 2023 Aug 7;11:e15621. doi: 10.7717/peerj.15621 (PMC10414028; doi:10.7717/peerj.15621)
Supplement: Supplemental Information 6 [file peerj-11-15621-s006.docx]

# Supplementary Tables

**Table S1**. Clinical information of samples in the Four cohorts.

| **Characteristic** | **GSE72094( N=386 )** | **GSE31210( N=226 )** | **GSE30219( N=83 )** | **TCGA( N=503 )** |
| --- | --- | --- | --- | --- |
| **Age, median**  **(interquartile range)** | 66 ( 59 to 72 ) | 61 ( 55 to 65 ) | 61.14 (52.04 to 62.05) | 70 ( 64 to 76.75 ) |
| **Gender (%)** |  |  |  |  |
| Female | 218 (56%) | 121 (54%) | 18 (22%) | 268 (53%) |
| Male | 168 (44%) | 105 (46%) | 65 (78%) | 235 (47%) |
| **Smoking (%)** |  |  |  |  |
| Ever | 291 (91%) | 111 (49%) |  | 346 (69%) |
| Never | 30 (9%) | 115 (51%) |  | 157 (31%) |
| **T category (%)** |  |  |  |  |
| T1&T2 |  |  | 81 (98%) | 438 (87%) |
| T3&T4 |  |  | 2 (2%) | 65 (13%) |
| **N category (%)** |  |  |  |  |
| N0 |  |  | 80 (96%) | 328 (65%) |
| N+ |  |  | 3 (4%) | 174 (35%) |
| Unknown |  |  |  | 1 (0%) |
| **M category (%)** |  |  |  |  |
| M0 |  |  | 83 (100%) | 334 (66%) |
| M+ |  |  |  | 164 (33%) |
| Unknown |  |  |  | 5 (1%) |
| **Pathological stage (%)** |  |  |  |  |
| Stage I&II | 311 (82%) | 226 (100%) |  | 391 (78%) |
| Stage III&IV | 70 (18%) |  |  | 104 (21%) |
| Unknown |  |  |  | 8 (2%) |
| **Status (%)** |  |  |  |  |
| Alive | 277(72%) | 191 (85%) | 40 (48%) | 322 (64%) |
| Dead | 109(28%) | 35 (15%) | 43 (52%) | 181 (36%) |
| OS time(days),  median (interquartile range) | 831.5  ( 568.5 to 1022.75 ) | 1744.5  ( 1245.5 to 2049.5 ) | 2070  ( 855 to 3435 ) | 677  ( 434.5 to 1147.5 ) |

**Table S2**. qPCR Primer sequence.

| **Gene Name** | **Primer sequence** |
| --- | --- |
| SCT1 F' | TTGCCTCAACAGTGCTCTAC |
| STC1 R' | AGCGCTGTACAAGAAGGATTTA |
| PFKL F' | CACATGACGGAGAAGATGAAGA |
| PFKL R' | CTTGCCCTCTGATGAGTACAG |
| HK1 F' | GAAGATGGTCAGTGGCATGTA |
| HK1 R' | GGTGATCCGCCCTTCAAATA |
| PDK3 F' | CCAGAGCTGGAAGTTGAAGAA |
| PDK3 R' | ACAGATGTGAGGGCACATAAA |
| SLC2A1 F' | CTGGGCAAGTCCTTTGAGAT |
| SLC2A1 R' | GTGACACTTCACCCACATACA |
| XPNPEP1 F' | TTGATGGTGACCGCATAGAC |
| XPNPEP1 R' | GATGCACCTGGATCCTGTATT |

**Table S3**. siRNA sequence.

| **Gene Name** | **Sense(5'-3')** | **Antisense(5'-3')** |
| --- | --- | --- |
| Si SLC2A1-1 | CAGCUACCCUGGAUGUCCUTT | AGGACAUCCAGGGUAGCUGTT |
| Si SLC2A1-2 | CAUCAACGCUGUCUUCUAUTT | AUAGAAGACAGCGUUGAUGTT |
| Si XPNPEP1 | GAUAGAUGUGGAUUCUCUUTT | AAGAGAAUCCACAUCUAUCTT |
| Si XPNPEP1 | UGAUAGAUGUGGAUUCUCUTT | AGAGAAUCCACAUCUAUCATT |

**Table S4.** Univariate and Multivariate Cox model regression analysis of the signature and clinical information with lung adenocarcinoma (LUAD) survival.

| **Variables** |  | **GSE72094** | | |  | **TCGA** | | |  | **GSE31210** | | |  | **GSE30219** | | |  |
| --- | --- | --- | --- | --- | --- | --- | --- | --- | --- | --- | --- | --- | --- | --- | --- | --- | --- |
|  |  | HR | 95%CI | *P*-value |  | HR | 95%CI | *P* -value |  | HR | 95%CI | *P* -value |  | HR | 95%CI | *P*- value |  |
| **Univariable Cox Regression** |  |  |  |  |  |  |  |  |  |  |  |  |  |  |  |  |  |
| Age | <65 vs.≥65 | 0.81 | 0.57-1.15 | 0.246 |  | 0.9 | 0.73-1.11 | 0.331 |  | 0.51 | 0.32-0.82 | 0.006 |  | 0.65 | 0.42-1.02 | 0.059 |  |
| Gender | Male vs. Female | 1.57 | 1.15-2.13 | 0.004 |  | 1.06 | 0.86-1.31 | 0.564 |  | 1.34 | 0.84-2.15 | 0.219 |  | 1.08 | 0.63-1.86 | 0.784 |  |
| Smoking | Never vs. Ever | 0.85 | 0.47-1.53 | 0.584 |  | 1.05 | 0.84-1.31 | 0.698 |  | 0.71 | 0.44-1.13 | 0.15 |  | - | - | - |  |
| Pathologic stage | Stage III&IV vs. Stage I&II | 2.25 | 1.63-3.11 | 0.001 |  | 1.93 | 1.54-2.41 | 0.001 |  | - | - | - |  | - | - | - |  |
| Pathologic T stage | T3&T4 vs. T1&T2 | - | - | - |  | 1.77 | 1.36-2.32 | 0.001 |  | - | - | - |  | 0.85 | 0.21-3.5 | 0.824 |  |
| Pathologic N stage | N0 vs. N+ | - | - | - |  | 0.52 | 0.42-0.65 | 0.001 |  | - | - | - |  | 1.17 | 0.43-3.21 | 0.762 |  |
| Pathologic M stage | M0 vs. M+ | - | - | - |  | 1.02 | 0.81-1.28 | 0.854 |  | - | - | - |  | - | - | - |  |
| EGFR mutation status | Wildtype vs. Mutant | 2.65 | 1.17-5.98 | 0.019 |  | - | - | - |  | - | - | - |  | - | - | - |  |
| KRAS mutation status | Wildtype vs. Mutant | 0.84 | 0.61-1.14 | 0.265 |  | - | - | - |  | - | - | - |  | - | - | - |  |
| STK11 mutation status | Wildtype vs. Mutant | 1.19 | 0.76-1.87 | 0.445 |  | - | - | - |  | - | - | - |  | - | - | - |  |
| TP53 mutation status | Wildtype vs. Mutant | 0.87 | 0.62-1.21 | 0.404 |  | - | - | - |  | - | - | - |  | - | - | - |  |
| Signature | Low-risk vs. High-risk | 0.33 | 0.23-0.47 | 0.001 |  | 0.79 | 0.64-0.98 | 0.029 |  | 0.45 | 0.26-0.76 | 0.003 |  | 0.58 | 0.37-0.9 | 0.014 |  |
|  |  |  |  |  |  |  |  |  |  |  |  |  |  |  |  |  |  |
| **Multivariable Cox Regression** |  |  |  |  |  |  |  |  |  |  |  |  |  |  |  |  |  |
| Age | <65 vs.≥65 | - | - | - |  | - | - | - |  | 0.49 | 0.3-0.8 | 0.004 |  | 0.62 | 0.4-0.96 | 0.034 |  |
| Gender | Male vs. Female | 1.72 | 1.26-2.35 | 0.001 |  | - | - | - |  | - | - | - |  | - | - | - |  |
| Smoking | Never vs. Ever | - | - | - |  | - | - | - |  | 0.69 | 0.43-1.12 | 0.133 |  | - | - | - |  |
| Pathologic stage | Stage III&IV vs. Stage I&II | 2.24 | 1.6-3.12 | 0.001 |  | 1.26 | 0.94-1.68 | 0.118 |  | - | - | - |  | - | - | - |  |
| Pathologic T stage | T3&T4 vs. T1&T2 | - | - | - |  | 1.41 | 1.04-1.9 | 0.026 |  | - | - | - |  | - | - | - |  |
| Pathologic N stage | N0 vs. N+ | - | - | - |  | 0.6 | 0.47-0.77 | 0 |  | - | - | - |  | - | - | - |  |
| Pathologic M stage | M0 vs. M+ | - | - | - |  | - | - | - |  | - | - | - |  | - | - | - |  |
| EGFR mutation status | Wildtype vs. Mutant | 1.96 | 0.86-4.44 | 0.108 |  | - | - | - |  | - | - | - |  | - | - | - |  |
| KRAS mutation status | Wildtype vs. Mutant | - | - | - |  | - | - | - |  | - | - | - |  | - | - | - |  |
| STK11 mutation status | Wildtype vs. Mutant | - | - | - |  | - | - | - |  | - | - | - |  | - | - | - |  |
| TP53 mutation status | Wildtype vs. Mutant | - | - | - |  | - | - | - |  | - | - | - |  | - | - | - |  |
| Signature | Low-risk vs. High-risk | 0.36 | 0.25-0.52 | 0.001 |  | 0.83 | 0.67-1.03 | 0.045 |  | 0.47 | 0.27-0.8 | 0.006 |  | 0.56 | 0.36-0.86 | 0.009 |  |
|  |  |  |  |  |  |  |  |  |  |  |  |  |  |  |  |  |  |

**Table S5.** C-index of Four cohorts.

| **Cohorts** | **C-index** | **low 95%CI** | **up 95%CI** |
| --- | --- | --- | --- |
| GSE72094 | 0.675 | 0.653 | 0.697 |
| GSE30219 | 0.609 | 0.569 | 0.649 |
| GSE31210 | 0.65 | 0.614 | 0.686 |
| TCGA-LUAD | 0.564 | 0.543 | 0.585 |

**Table S6.** Significantly enriched pathways between the high-risk and low-risk groups.

| **ID** | **Description** | **EnrichmentScore** | **NES** | **P-value** |
| --- | --- | --- | --- | --- |
| hsa05203 | Viral carcinogenesis | -0.397749806 | -1.519622661 | 0.004201681 |
| hsa03010 | Ribosome | -0.424376415 | -1.521459694 | 0.006396588 |
| hsa00563 | Glycosylphosphatidylinositol (GPI)-anchor biosynthesis | 0.606531556 | 1.612949946 | 0.010695187 |
| hsa03060 | Protein export | 0.600531747 | 1.582720743 | 0.019264448 |
| hsa04510 | Focal adhesion | -0.343934453 | -1.320206081 | 0.023206751 |
| hsa00020 | Citrate cycle (TCA cycle) | 0.5352258 | 1.472200593 | 0.027573529 |
| hsa04630 | JAK-STAT signaling pathway | -0.366211546 | -1.350700403 | 0.034408602 |
| hsa00592 | alpha-Linolenic acid metabolism | -0.572435687 | -1.525779602 | 0.034802784 |
| hsa04950 | Maturity onset diabetes of the young | 0.561318878 | 1.492715827 | 0.040998217 |
| hsa00260 | Glycine, serine, and threonine metabolism | 0.49756281 | 1.432317267 | 0.041742287 |
| hsa00760 | Nicotinate and nicotinamide metabolism | 0.515072394 | 1.416766315 | 0.045955882 |

**Table S7.** Five drugs with the connectivity scores in A549 in the CMap analysis.

| **Drug** | **Dose** | **Time** | **Cell** | **Connectivity score** |
| --- | --- | --- | --- | --- |
| Paclitaxel | 10 uM | 24h | A549 | -0.92 |
| Vinorelbine | 10 uM | 24h | A549 | -0.77 |
| Gefitinib | 10 uM | 24h | A549 | 0.09 |
| Erlotinib | 2.22 uM | 24h | A549 | 0.58 |
| Docetaxel | 10 uM | 24h | A549 | 0.83 |
